# Effect of Environmental and Lifestyle Factors on Hypertension: Shimane COHRE Study

**DOI:** 10.1371/journal.pone.0049122

**Published:** 2012-11-09

**Authors:** Tsuyoshi Hamano, Yoshinari Kimura, Miwako Takeda, Masayuki Yamasaki, Minoru Isomura, Toru Nabika, Kuninori Shiwaku

**Affiliations:** 1 Center for Community-Based Health Research and Education (COHRE), Organization for the Promotion of Project Research, Shimane University, Izumo, Japan; 2 Department of Environmental and Preventive Medicine, Shimane University School of Medicine, Izumo, Japan; 3 Department of Geography, Graduate School of Literature and Human Sciences, Osaka City University, Osaka, Japan; 4 Department of Functional Pathology, Shimane University School of Medicine, Izumo, Japan; Fundación para la Prevención y el Control de las Enfermedades Crónicas No Transmisibles en América Latina (FunPRECAL), Argentina

## Abstract

**Background:**

In recent years there has been increasing evidence of an association between residential remoteness and hypertension (HTN); however, no study has examined the effects of residential remoteness-lifestyle associations on HTN. The objective of this study was to evaluate the effects of residential remoteness, as measured by road network distance and elevation, and lifestyle associations, including access to daily products as a measure of car use, on HTN in a rural region in Japan.

**Method:**

This is a cross-sectional population based study. We analyzed data from the Shimane COHRE study conducted from 2006 to 2009 in the rural mountainous regions of Japan. After excluding missing data, we conducted a logistic regression analysis of the data for 1,348 individuals and examined the effects of residential remoteness and lifestyle associations, including road network distance, elevation and access to daily products as a measure of car use, on the prevalence of HTN.

**Principal Findings:**

In participants without access to car use, the odds ratios for self-reported HTN (i.e. taking antihypertensive medication) were significantly increased in those living in moderate (odds ratio (OR): 2.21, 95% confidence interval (CI): 1.19–4.08) and far (OR: 2.55, 95% CI: 1.00–6.51) road distances, whereas there were no significant associations in participants with access to car use. There were no significant associations between elevation and HTN for participants either with or without access to car transportation.

**Conclusions:**

Our findings show that specific residential remoteness-hypertension associations vary according to access to daily products as a measure of car use in a rural mountainous area of Japan. These results advance the understanding and importance of considering residential environment, “where people live,” in establishing health policy.

## Introduction

Hypertension (HTN) is a major public health problem and worldwide prevalence has been estimated as high as 1 billion individuals [1]. Previous studies have found that lifestyle-related factors (e.g. obesity, excessive alcohol consumption and physical inactivity) [2], low socioeconomic status (e.g. education and occupation) [3], and genetic variants [4] are associated with HTN.

Over the past several years there have been efforts to examine the associations between characteristics of residential environments and HTN [5]–[9]. Most of these studies have focused on two broad factors: physical environment (e.g. walkability and environmental exposures) and social environment (e.g. socioeconomic deprivation and social capital) [10]. More recently research on the association between residential remoteness of the physical environment and HTN has gained momentum with increasing attention being paid to Geographic Information Systems (GIS) [10]–[12]. Among the previous studies investigating residential remoteness, road network distance between participant location and the population center has been estimated by GIS. The initial study looking at potential correlations between residential remoteness and HTN (conducted in a developing country) found that there was a significant inverse association between distance from a population center and blood pressure (BP) [11]. In addition, evidence from a study in a developed country showed that residential remoteness, measured by road network distance, was associated with a higher risk of HTN [12]. To our knowledge, no previous study has examined the effects of residential remoteness-lifestyle associations on HTN.

Transportation is an important lifestyle factor determining health-promoting behaviors in rural areas [13], [14]. Given that public transportation networks in rural areas are often poor, the availability of healthcare utilization may be limited [13], [15] as well as food availability [16]. As such, it has been hypothesized that access to car transport may be a key factor in the association between residential remoteness and HTN.

In the present study, the elevation of each participant’s residential address was estimated by GIS as another proxy of residential remoteness. Since areas of higher elevation often have poor or no public transportation and food services are frequently limited also due to limits in transportation, people residing in such areas may consume more traditional Japanese food, which has a higher salt content compared to food generally consumed at lower residential elevations, suggesting that diets which are restricted due to limited food access (i.e. defined by no access to car transportation) may potentially effect BP. In addition, persons living in remote mountain regions also may have restricted access to healthcare services owing to poor public transportation. The objective of this study was to examine the effect of remoteness, defined by road network distance, elevation, and lifestyle, including availability of daily products as a measure of access to car transport, on HTN prevalence in a rural region in the mountainous of Japan.

## Materials and Methods

### Ethics Statement

The study protocol was approved by the ethics committee of the Shimane University School of Medicine in April 2006, and written informed consent was obtained from all participants.

### Data Collection

**Figure 1:**
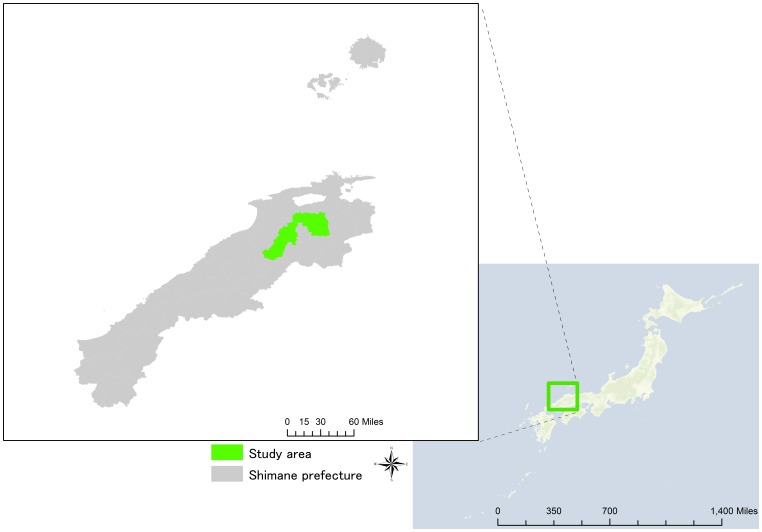
Map of study area.

Data was collected in a cross-sectional study conducted from 2006 to 2009 as a part of the Shimane COHRE Study, which was designed to examine the determinants of lifestyle-related diseases, including HTN. The Shimane COHRE study, conducted by Shimane University in Japan, was undertaken in collaboration with a health examination program conducted in 4 municipalities: the towns of Kakeya (since 2006), Mitoya (since 2007), Daito (since 2009), and Kamo (since 2009). These towns are located in rural mountainous regions in the eastern part of Shimane prefecture, Japan (**Figure 1**). The residents who live in these municipalities have two options for their health examination. One is examination in a group setting conducted at public health center and the other is examination in a private room setting at a medical institution. Our study was given permission to use data from the group setting examinations for this analysis.

### Measurements

HTN was defined according to the data obtained in face-to-face interviews conducted by trained staff [17], and seated BP measurements. The participants were asked the following question: “Are you currently under treatment for HTN?” In response, 362 participants reported that they were under treatment and were taking antihypertensive medication, 31 participants reported that they were under treatment for HTN without receiving medication, and 955 participants reported they were not under any treatment for HTN. The four HTN subgroups were defined as follows: (1) taking antihypertensive medication (**Model 1A**), (2) taking antihypertensive medication or under treatment without medication (**Model 1B**), (3) taking antihypertensive medication or BP≥140/90 mm Hg for systolic BP (SBP)/diastolic BP (DBP) (**Model 2A**), (4) taking antihypertensive medication or under treatment without medication or BP≥140/90 mm Hg for SBP/DBP (**Model 2B**).

We also considered the following variables in the analysis: age, sex (male, female), employment (agriculture, self-employed, other, or unemployed), body mass index (BMI), stress (currently feel stress = yes or do not currently feel stress = no), use of a car for daily transportation (non-car use or car use), alcohol consumption (non-drinker or drinker), smoking (non-smoker or smoker), physical activity (regularly engaged in physical activity = yes or did not regularly engage in physical activity = no), and use of medication treatment (dyslipidemia or diabetes).

### GIS Measurements

GIS were used to estimate road network distance and elevation, and for database queries. In this study we used ArcGIS 10.0 software from ESRI (Redlands, CA). For road network distance we estimated the distance between each participant’s actual residential location and the population center. The location of the Shimane prefectural government was defined as the population center [12]. Network analysis, which determined the shortest path between the participant locations and the population center, was performed on road networks, then the road network distance was divided into tertiles: 0–26,685.8 m = close distance; 26,685.9–38,350.6 m = moderate distance; and 38,350.7–68,070.1 m = far distance.

The elevation model for each participan’s residential location was developed by using the ready-to-use dataset of digital elevation models for GIS (ESRI Japan Corporation, Tokyo, JAPAN). Elevation was divided into tertiles: 0–52.0 m = low elevation; 53.0–187.0 m = middle elevation; and 188.0–522.0 m = high elevation. In addition, distance to the nearest bus stop was estimated on the road networks and divided as follows: 0–305.12 m = close distance and 305.13 m–5,199.84 m = far distance. Distance to the nearest clinic was estimated on the road networks as well. The median was used to characterize the two groups: 0–2,180.14 m = close distance and 2,180.15–19,883.31 m = far distance.

### Statistical Analyses

Descriptive statistics were calculated, and χ^2^ and t-tests were conducted to compare the characteristics between the car use and non-car use groups. All variables (road network distance, elevation, age, sex, employment, medication use, smoking, alcohol consumption, BMI, physical activity, stress, distance to the nearest bus stop, and distance to the nearest clinic) were entered into a logistic regression model to identify independent associations with HTN. In this study, four separate logistic regression models were created to derive adjusted odds ratio (OR) of the independent association of each predictor variable: (1) **Model 1A** definition of HTN (taking antihypertensive medication); (2) **Model 1B** definition of HTN (taking antihypertensive medication or under treatment for hypertension without medication); (3) **Model 2A** definition of HTN (taking antihypertensive medication or BP≥140/90 mm Hg for SBP/DBP); and (4) **Model 2B** definition of HTN (taking antihypertensive medication or under treatment without medication or BP≥140/90 mmHg for SBP/DBP). The present study involved 1,458 participants from 4 municipalities. After excluding the participants for whom data was missing we analyzed the data for 1,348 participants aged 40 to 74 years. p<0.05 was considered statistically significant. All statistical analyses were performed using SPSS for Windows (SPSS Japan, Tokyo, JAPAN).

## Results

The characteristics of study participants are shown in **Table 1**. Of these 954 used a car as their primary transportation in daily life. Non-car users were generally of relatively older age, female, unemployed, non-smoker, non-drinker, lower BMI, physically active, and with treatment for dyslipidemia. There were no significant differences between two groups relative to HTN.

**Table 1:** Characteristics of the study participants.

	Car use (n = 954)		Non car use (n = 394)		*p*
	n	(%) orMean (SD)	n	(%) orMean (SD)	
HTN					
Model 1A definition, %	245	(25.7)	117	(29.7)	0.13
Model 1B definition, %	267	(28.0)	126	(32.0)	0.14
Model 2A definition, %	406	(42.6)	178	(45.2)	0.37
Model 2B definition, %	418	(43.8)	181	(45.9)	0.47
Age, years (SD)	954	64.1 (7.13)	394	67.0 (5.91)	<0.01
Sex, % male	444	(46.5)	66	(16.8)	<0.01
Employment					<0.01
Agriculture, %	241	(25.3)	96	(24.4)	
Self-employed, %	172	(18.0)	39	(9.9)	
Other, %	221	(23.2)	61	(15.5)	
Unemployed, %	320	(33.5)	198	(50.3)	
Medication use					
Dyslipidemia, %	113	(11.8)	64	(16.2)	0.03
Diabetes, %	39	(4.1)	15	(3.8)	0.81
Smoking, %	102	(10.7)	14	(3.6)	<0.01
Alcohol consumption, %	466	(48.8)	98	(24.9)	<0.01
BMI, kg/m^2^ (SD)	954	22.7 (2.97)	394	22.2 (2.88)	<0.01
Physical activity, %	288	(30.2)	174	(44.2)	<0.01
Stress, %	431	(45.2)	184	(46.7)	0.61

The continuous variables are presented as mean (SD) and p-value (t-test). The discrete variables are presented as frequency (%) and p-value (χ^2^ -test). In **Model 1A**, HTN is defined by self-report (taking antihypertensive medication). In **Model 1B**, HTN is defined by self-report (taking antihypertensive medication or under treatment for HTN without medication). In **Model 2A**, HTN is defined by self-report and measuring BP (taking antihypertensive medication or BP≥140/90 mm Hg for SBP/DBP). In **Model 2B**, HTN is defined by self-report and measuring BP (taking antihypertensive medication or under treatment for HTN without medication or BP≥140/90 mm Hg for SBP/DBP).

**HTN**, hypertension; **BMI**, body mass index.

**Table 2** provides the results of the adjusted OR and 95% confidence interval (CI) in non-car users. Using the **Model 1A** definition of HTN (taking antihypertensive medication), the odds ratios (ORs) for HTN were 2.21 (95% CI: 1.19–4.08, *P*<0.05) and 2.55 (95% CI: 1.00–6.51, *P*<0.05) in moderate and far distances, respectively. As to elevation, the ORs for HTN were decreased compared with low elevation (moderate elevation: OR = 0.73, 95% CI: 0.38–1.43; high elevation: OR = 0.50, 95% CI: 0.19–1.28); however, these results were not statistically significant. Age (OR = 1.06, 95% CI: 1.01–1.12), medication use for dyslipidemia (OR = 4.13, 95% CI: 2.24–7.58) and BMI (OR = 1.13, 95% CI: 1.04–1.24) were significantly associated with hypertensive status. Using the **Model 1B** definition of HTN (taking antihypertensive medication or under treatment for HTN without medication), the ORs for HTN were 2.29 (95% CI: 1.26–4.18, *P*<0.01) and 2.73 (95% CI: 1.09–6.82, *P*<0.05) in moderate and far distances, respectively. Elevation was not associated significantly with hypertensive status (moderate elevation: OR = 0.74, 95% CI: 0.39–1.43; high elevation: OR = 0.53, 95% CI: 0.21–1.32). Age (OR = 1.06, 95% CI: 1.01–1.11), medication use for dyslipidemia (OR = 3.79, 95% CI: 2.07–6.93) and BMI (OR = 1.11, 95% CI: 1.02–1.21) were significantly associated with hypertensive status. Using the **Model 2A** (taking antihypertensive medication or BP≥140/90 mmHg for SBP/DBP) and **2B** (taking antihypertensive medication or under treatment for HTN without medication or BP≥140/90 mmHg for SBP/DBP) definitions of HTN, road network distance were not significantly associated with hypertensive status. Regarding elevation, the ORs were decreased; however, they were not associated significantly with hypertensive status. Age (Model 2A OR = 1.04, 95% CI: 1.00–1.09; Model 2B OR = 1.04, 95% CI: 1.00–1.08), medication use for dyslipidemia (Model 2A OR = 2.80, 95% CI: 1.52–5.15; Model 2B OR = 2.90, 95% CI: 1.57–5.35) and BMI (Model 2A OR = 1.15, 95% CI: 1.06–1.25; Model 2B OR = 1.15, 95% CI: 1.06–1.25) were significantly associated with hypertensive status.

**Table 2:** Multivariate logistic regression models of HTN (non-car use, n = 394).

	Self-reported HTN								Self-reported HTN and BP measurements							
	Model 1A				Model 1B				Model 2A				Model 2B			
	aOR	95%CI			aOR	95%CI			aOR	95%CI			aOR	95%CI		
Road network distance																
Close distance (reference)																
Moderate distance	2.21	1.19	–	4.08	2.29	1.26	–	4.18	1.37	0.80	–	2.36	1.43	0.83	–	2.45
Far distance	2.55	1.00	–	6.51	2.73	1.09	–	6.82	1.20	0.52	–	2.75	1.27	0.55	–	2.91
Elevation																
Low elevation (reference)																
Moderate elevation	0.73	0.38	–	1.43	0.74	0.39	–	1.43	0.84	0.47	–	1.51	0.86	0.48	–	1.54
High elevation	0.50	0.19	–	1.28	0.53	0.21	–	1.32	0.70	0.30	–	1.62	0.72	0.31	–	1.65
Age	1.06	1.01	–	1.12	1.06	1.01	–	1.11	1.04	1.00	–	1.09	1.04	1.00	–	1.08
Sex	1.44	0.63	–	3.28	1.12	0.50	–	2.52	1.50	0.73	–	3.08	1.36	0.67	–	2.79
Employment																
Agriculture (reference)																
Self-employed	1.86	0.70	–	4.97	1.63	0.63	–	4.19	2.33	0.98	–	5.50	1.97	0.83	–	4.63
Other	2.19	0.93	–	5.15	1.81	0.79	–	4.15	2.35	1.11	–	4.99	2.02	0.96	–	4.24
Unemployed	2.24	1.15	–	4.35	1.88	1.00	–	3.56	2.28	1.27	–	4.08	1.95	1.10	–	3.46
Medication use																
Dyslipidemia	4.13	2.24	–	7.58	3.79	2.07	–	6.93	2.80	1.52	–	5.15	2.90	1.57	–	5.35
Diabetes	1.27	0.39	–	4.07	1.60	0.51	–	5.01	1.60	0.48	–	5.23	1.55	0.47	–	5.07
Smoking	0.16	0.01	–	1.49	0.17	0.01	–	1.49	0.48	0.13	–	1.79	0.49	0.13	–	1.84
Alcohol consumption	0.93	0.47	–	1.83	1.04	0.54	–	2.01	0.76	0.42	–	1.38	0.80	0.44	–	1.44
BMI	1.13	1.04	–	1.24	1.11	1.02	–	1.21	1.15	1.06	–	1.25	1.15	1.06	–	1.25
Physical activity	1.12	0.67	–	1.86	1.12	0.68	–	1.84	1.04	0.66	–	1.64	1.01	0.64	–	1.58
Stress	1.07	0.65	–	1.76	1.10	0.68	–	1.79	0.93	0.60	–	1.45	0.91	0.58	–	1.41
Distance to the nearest bus stop	1.54	0.90	–	2.61	1.44	0.86	–	2.42	1.49	0.92	–	2.40	1.46	0.91	–	2.36
Distance to the nearest clinic	1.38	0.79	–	2.41	1.23	0.72	–	2.13	1.08	0.65	–	1.79	1.05	0.64	–	1.73

Reference categories are female, non-smoking, non-drinker, did not engage in physical activity regularly, did not feel stress, close distance to the nearest bus stop, and close distance to the nearest clinic.

In **Model 1A**, HTN is defined by self-report (taking antihypertensive medication). In **Model 1B**, HTN is defined by self-report (taking antihypertensive medication or under treatment for HTN without medication). In **Model 2A**, HTN is defined by self-report and measuring BP (taking antihypertensive medication or BP≥140/90 mm Hg for SBP/DBP). In **Model 2B**, HTN is defined by self-report and measuring BP (taking antihypertensive medication or under treatment for HTN without medication or BP≥140/90 mm Hg for SBP/DBP).

**HTN**, hypertension; **aOR**, adjusted odds ratio; **CI**, confidence interval; **BP**, blood pressure; **SBP**, systolic blood pressure; **DBP**, diastolic blood pressure; **BMI**, body mass index.

**Table 3** provides the results of the adjusted OR and 95% CI in car users. Participants in the **Model 1A** subgroup showed no significant associations between road network distance and hypertensive status (moderate distance: OR = 0.95, 95% CI: 0.62–1.46; far distance: OR = 0.95, 95% CI: 0.55–1.62). With regard to elevation, the ORs for HTN were increased (moderate elevation: OR = 1.19, 95% CI: 0.77–1.84; high elevation: OR = 1.64, 95% CI: 0.92–2.94), however, these results were not statistically significant. Age (OR = 1.08, 95% CI: 1.05–1.11), medication use for dyslipidemia (OR = 3.44, 95% CI: 2.20–5.38) and BMI (OR = 1.17, 95% CI: 1.11–1.24) were significantly associated with hypertensive status. Participants in the **Model 1B** subgroup demonstrated no significant associations between remoteness, measured by road network distance, elevation, and hypertensive status. Age (OR = 1.08, 95% CI: 1.05–1.11), medication use for dyslipidemia (OR = 2.85, 95% CI: 1.82–4.46) and BMI (OR = 1.19, 95% CI: 1.12–1.25) were significantly associated with hypertensive status. Furthermore, similar patterns were observed in participants from both the **Model 2A and 2B** subgroups with no statistically significant associations between remoteness, measured by road network distance, elevation, and risk of HTN. Age (Model 2A OR = 1.06, 95% CI: 1.03–1.08; Model 2B OR = 1.06, 95% CI: 1.03–1.08), medication use for dyslipidemia (Model 2A OR = 2.19, 95% CI: 1.39–3.43; Model 2B OR = 2.05, 95% CI: 1.30–3.21), alcohol consumption (Model 2A OR = 1.50, 95% CI: 1.06–2.14; Model 2B OR = 1.52, 95% CI: 1.07–2.16) and BMI (Model 2A OR = 1.24, 95% CI: 1.17–1.30; Model 2B OR = 1.24, 95% CI: 1.18–1.31) were significantly associated with hypertensive status.

**Table 3:** Multivariate logistic regression models of HTN (car use, n = 954).

	Self-reported HTN								Self-reported HTN and BP measurements							
	Model 1A				Model 1B				Model 2A				Model 2B			
	aOR	95%CI			aOR	95%CI			aOR	95%CI			aOR	95%CI		
Road network distance																
Close distance (reference)																
Moderate distance	0.95	0.62	–	1.46	1.12	0.74	–	1.70	0.96	0.66	–	1.38	1.03	0.71	–	1.49
Far distance	0.95	0.55	–	1.62	1.18	0.70	–	2.00	0.98	0.60	–	1.58	1.08	0.67	–	1.76
Elevation																
Low elevation (reference)																
Moderate elevation	1.19	0.77	–	1.84	1.10	0.72	–	1.69	0.89	0.61	–	1.30	0.88	0.60	–	1.29
High elevation	1.64	0.92	–	2.94	1.56	0.88	–	2.75	1.02	0.60	–	1.72	1.01	0.60	–	1.71
Age	1.08	1.05	–	1.11	1.08	1.05	–	1.11	1.06	1.03	–	1.08	1.06	1.03	–	1.08
Sex	1.37	0.88	–	2.12	1.30	0.85	–	1.98	1.15	0.79	–	1.69	1.19	0.81	–	1.74
Employment																
Agriculture (reference)																
Self-employed	1.46	0.87	–	2.44	1.31	0.79	–	2.16	1.52	0.95	–	2.41	1.39	0.88	–	2.21
Other	1.11	0.67	–	1.82	1.07	0.66	–	1.73	0.99	0.64	–	1.54	0.97	0.63	–	1.50
Unemployed	1.29	0.83	–	1.99	1.20	0.78	–	1.82	1.60	1.08	–	2.37	1.51	1.02	–	2.22
Medication use																
Dyslipidemia	3.44	2.20	–	5.38	2.85	1.82	–	4.46	2.19	1.39	–	3.43	2.05	1.30	–	3.21
Diabetes	1.59	0.77	–	3.28	1.81	0.89	–	3.68	1.72	0.84	–	3.51	1.82	0.89	–	3.74
Smoking	0.80	0.45	–	1.42	0.76	0.44	–	1.33	0.79	0.49	–	1.30	0.78	0.48	–	1.27
Alcohol consumption	1.23	0.82	–	1.85	1.27	0.86	–	1.89	1.50	1.06	–	2.14	1.52	1.07	–	2.16
BMI	1.17	1.11	–	1.24	1.19	1.12	–	1.25	1.24	1.17	–	1.30	1.24	1.18	–	1.31
Physical activity	0.88	0.62	–	1.26	0.84	0.59	–	1.19	0.80	0.58	–	1.10	0.82	0.59	–	1.12
Stress	1.15	0.82	–	1.60	1.18	0.85	–	1.63	1.04	0.77	–	1.39	1.12	0.84	–	1.51
Distance to the nearest bus stop	1.04	0.73	–	1.47	1.11	0.79	–	1.55	1.01	0.74	–	1.37	1.04	0.77	–	1.42
Distance to the nearest clinic	1.10	0.77	–	1.58	1.03	0.73	–	1.47	1.05	0.76	–	1.45	1.02	0.74	–	1.40

Reference categories are female, non-smoking, non-drinker, did not engage in physical activity regularly, did not feel stress, close distance to the nearest bus stop, and close distance to the nearest clinic.

In **Model 1A**, HTN is defined by self-report (taking antihypertensive medication). In **Model 1B**, HTN is defined by self-report (taking antihypertensive medication or under treatment for HTN without medication). In **Model 2A**, HTN is defined by self-report and measuring BP (taking antihypertensive medication or BP≥140/90 mm Hg for SBP/DBP). In **Model 2B**, HTN is defined by self-report and measuring BP (taking antihypertensive medication or under treatment for HTN without medication or BP≥140/90 mm Hg for SBP/DBP).

**HTN**, hypertension; **aOR**, adjusted odds ratio; **CI**, confidence interval; **BP**, blood pressure; **SBP**, systolic blood pressure; **DBP**, diastolic blood pressure; **BMI**, body mass index.

## Discussion

Our findings suggest that the association between residential remoteness and HTN may vary by the availability of transportation for daily activity in rural regions. In addition, this study extends the hypothesis connecting local life environment and level of health, categorized as “location where people live,” to include applications in rural settings. Although previous studies have focused on road network distance as a proxy for residential remoteness (horizontal distance between subject and population center) [11], this study included elevation (vertical distance between participants and the reference point) determined by GIS methodology in order to evaluate geographical differences potentially related to hypertensive status. The only other study we found in the literature dealing with this vertical perspective reported associations between residential environments, as measured by remotely sensed data for land cover/land use, and HTN [9]. The authors developed a land-use map based on the earth’s surface data gathered by the Landsat satellite. The importance of this inclusion is that in the rural setting there are a variety of environmental attributes (i.e. urbanized, typical rural, and mixture of urban and rural) compared with urban areas, and the rural trends might not be apparent in one-dimensional linear patterns measuring only distance from population centers.

As expected, this study found that for non-car users, there were statistically significantly higher risks of HTN for those living in more distant locations. The mean nearest distances to public transportation in the moderate and far distance groups (463.6±662.9 m, 1,193.2±1,330.7 m, respectively) were significantly farther than in the close distance group (233.1±175.8 m; *P*<0.01). These results suggest that lack of access to transportation may be important in predicting/determining hypertensive status in rural regions. One explanation for this is that lack of access to transportation may lead to lower utilization of health services (i.e. heath education programs) as well as fewer regular health maintenance check-ups [13]. For example, the mean distances to the nearest clinic in the moderate and far distance groups (3,195.9±3,492.0 m and 5,915.9±5,770.2 m, respectively) were significantly farther than in the close distance group (1,301.5±1,420.5 m; *P*<0.01). At the same time, the mean distances to the nearest hospital in far distance groups also were significantly farther than in those of the close distance group (data not shown). As such these results suggest that greater distance may impose a significant barrier to access for health services. Further studies are needed to provide a more comprehensive understanding of the associations between availability to transportation and access to health-promoting services in this and/or other rural regions.

A potentially confounding variable may be responsible for the apparent lack of a consistent pattern. In the participants having group setting examinations, BP measurement may increase bias toward higher BP compared with routine measurements taken at home. Because in the public center, study participants have their health checkups in the same room (separate rooms are not available for each person) which this may promote anxiety, nervousness or the other stress-related response, due to the setting and lack of quiet. Therefore, further studies are required to investigate this consideration as well.

We found no significant associations between elevation and hypertensive status. We hypothesized that those who live at a higher elevation and were non-car users would have a higher OR for HTN. One possible explanation for these alternate findings is that the topographical variance in elevation in the region examined is not great (mean, 134.1±109.2 m). For example, previous study in Saudi Arabia found that subjects living at high (3,150 m) had a higher risk of HTN than those living at low (500 m) [18]. On the other hand, another study conducted in central Asia found that HTN is more frequent in subjects living at low (600 m) than those who live at high (3,200 m) [19]. This evidence indicates need for careful interpretation of the effect of remoteness taking into account its context, such as culture, economic growth, and traditional back ground. Another possible reason for the lack of association is that the participants living at higher elevations may incorporate higher exercise intensity in their activity compared to those who live at lower elevations, thus the local environment may promote greater physical activity that may have a lowering affect on BP.

To our knowledge, the present study is the first to focus on both horizontal and vertical distances from a reference point, which advances the literature by showing that residential remoteness is associated with hypertensive status. This potential influence of residential environment on HTN may have a complex pathogenesis. This is because HTN is a multifactorial disease, and it is hypothesized that the effect of residential environment may vary depending on individual characteristics. A possible fruitful area for research is the gene–environment interaction. Although few studies have shown an effect of the gene–lifestyle interaction on HTN [20], [21], the synergistic effect on HTN remains unclear. While addressing these challenges was beyond the scope of our paper, we consider this subject a potentially important issue for further exploration.

In this study, the participants in the car user group were relatively young, more likely to be employed, and were taking less treatment for chronic disease, so this potential confounder needs further evaluation as well. Even so, as the younger participants age, they may not be able to rely on access to a car as their main form of transportation. That being the case, particularly aging participants who live more remotely may experience restricted access or barriers to health services. As such, research on residential remoteness-lifestyle associations may be of great importance for HTN prevention. Although the scope of our study did not allow us to expand on this point, we consider it to be a potentially important issue for health policy in rural regions.

This study has notable strengths. An important factor is that we measured road network distance between the participants’ actual addresses and the population center to capture residential remoteness. As such, our approach may contain less measurement ‘noise’ than would be generated using straight-line distance calculations, particularly in that the road network is more complex in mountainous regions. In addition, to the best of our knowledge, our study is the first to examine the association between elevation and HTN using GIS methodology. Furthermore, we demonstrate the effects of residential remoteness-lifestyle associations on hypertensive status for the first time. Additional studies using a geographic perspective are desirable, would reinforce these results and improve robustness of this data (if the outcomes are replicated), and may be effective in helping to establish health policies.

The present study also has certain limitations. This data is not an equitably representative sample. In particular, most participants had reached retirement age and the majority of participants were female. A selection bias caused by non-respondents in each town also was present and may have influenced the association between residential remoteness and HTN. Caution is therefore warranted in over-interpreting these findings. In addition, our data did not allow us to assess other important risk factors for HTN, such as income, education, diet, and family history. Confines of this study did not allow us to consider food accessibility and eating habits as well. Furthermore, we used a self-reported process to define the presence of HTN and also the BP measures taken at a public center may increase bias toward a higher measurement. These methodological considerations may affect the results. Besides, since our study was based on self-reported, interviewer bias may result in misclassification. However, we have attempted to reduce this potential bias by training before conducting the survey. Finally, the present study used a cross-sectional design, which did not allow establishment of the temporal order of causality.

In conclusion, our findings show that the specific associations between remoteness and hypertensive status differed by availability of daily products as a definition of access to car transport in a rural region in the mountainous of Japan. These results advance the more understanding and importance for considering residential environment and “where people live” when establishing health policies.
